# To the Root of the Curl: A Signature of a Recent Selective Sweep Identifies a Mutation That Defines the Cornish Rex Cat Breed

**DOI:** 10.1371/journal.pone.0067105

**Published:** 2013-06-27

**Authors:** Barbara Gandolfi, Hasan Alhaddad, Verena K. Affolter, Jeffrey Brockman, Jens Haggstrom, Shannon E. K. Joslin, Amanda L. Koehne, James C. Mullikin, Catherine A. Outerbridge, Wesley C. Warren, Leslie A. Lyons

**Affiliations:** 1 Department of Population Health and Reproduction, School of Veterinary Medicine, University of California - Davis, Davis, California, United States of America; 2 Department of Pathology, Microbiology, Immunology, School of Veterinary Medicine, University of California - Davis, Davis, California, United States of America; 3 Hill’s Pet Nutrition Center, Topeka, Kansas, United States of America; 4 Department of Clinical Sciences, Faculty of Veterinary Medicine and Animal Science, Swedish University of Agricultural Sciences, Uppsala, Sweden; 5 Comparative Genomics Unit, Genome Technology Branch, National Human Genome Research Institute, National Institutes of Health, Bethesda, Maryland, United States of America; 6 Department of Veterinary Medicine & Epidemiology, School of Veterinary Medicine, University of California - Davis, Davis, California, United States of America; 7 The Genome Institute, Washington University School of Medicine, St. Louis, Missouri, United States of America; University of Iceland, Iceland

## Abstract

The cat (*Felis silvestris catus)* shows significant variation in pelage, morphological, and behavioral phenotypes amongst its over 40 domesticated breeds. The majority of the breed specific phenotypic presentations originated through artificial selection, especially on desired novel phenotypic characteristics that arose only a few hundred years ago. Variations in coat texture and color of hair often delineate breeds amongst domestic animals. Although the genetic basis of several feline coat colors and hair lengths are characterized, less is known about the genes influencing variation in coat growth and texture, especially rexoid – curly coated types. Cornish Rex is a cat breed defined by a fixed recessive curly coat trait. Genome-wide analyses for selection (d_i_, Tajima’s D and nucleotide diversity) were performed in the Cornish Rex breed and in 11 phenotypically diverse breeds and two random bred populations. Approximately 63K SNPs were used in the analysis that aimed to localize the locus controlling the rexoid hair texture. A region with a strong signature of recent selective sweep was identified in the Cornish Rex breed on chromosome A1, as well as a consensus block of homozygosity that spans approximately 3 Mb. Inspection of the region for candidate genes led to the identification of the *lysophosphatidic acid receptor 6* (*LPAR6*). A 4 bp deletion in exon 5, c.250_253_delTTTG, which induces a premature stop codon in the receptor, was identified via Sanger sequencing. The mutation is fixed in Cornish Rex, absent in all straight haired cats analyzed, and is also segregating in the German Rex breed. *LPAR6* encodes a G protein-coupled receptor essential for maintaining the structural integrity of the hair shaft; and has mutations resulting in a wooly hair phenotype in humans.

## Introduction

Phenotypic traits under strong artificial selection within cat breeds vary from body types, muzzle shape, tail length to aesthetically pleasant traits, such as hair color, length and texture. Hair represents one of the defining characteristic of mammals. Hair provides body temperature regulation, protection from environmental elements, and adaptive advantages of camouflage, as well as often having aesthetic value to humans. The hair follicle has a highly complex structure with eight distinct cell layers, in which hundreds of gene products play a key role in the hair cycle maintenance [1], [2]. In the past decade, numerous genes expressed in the hair follicle have been identified and mutations in some of these genes have been shown to underlie hereditary hair diseases in humans and other mammals [3]. Hereditary hair diseases in mammals show diverse hair phenotypes, such as sparse or short hairs (hypotrichosis), excessive or elongated hairs (hypertrichosis), and hair shaft anomalies, creating rexoid/woolly hairs [3]–[12].

Causative genes for the diseases encode various proteins with different functions, such as structural proteins, transcription factors, and signaling molecules. Mutations within structural proteins, such as epithelial and hair keratins, are often associated with hair disease. To date, mutations in several hair keratin genes underlined two hereditary hair disorders: monilethrix, characterized by fragile hair shafts (in *KRT81, KRT83* or *KRT86* [13], [14]), and pure hair and nail ectodermal dysplasia in *KRT85* (PHNED) [15]. In addition to hair keratins, epithelial keratins such as *KRT24 - 28* and *KRT71 - 74* are predominantly expressed in the hair follicle (HF) [16], [17]. Mutations within the *KRT71* keratin are associated with curly-wavy phenotypes in mice [18], dog [6], cat [4], rat [19] and human [8], while mutations within the *KRT74* gene are associated with woolly hair and hypotrichosis in humans [9], [20]. Primary HF formation is controlled by three distinct signaling pathways (Wnt, Hhs, and Adar) interconnected in a signaling cascade. The WNT/β-catenin signaling is critical for the initiation of the HF development [21], [22] and can be inhibited by *APCDD1* [23]. A mutation within *APCDD1* is associated with the autosomal dominant hereditary hypotrichosis simplex (HHS), characterized by progressive loss of hair [24], [25]. Moreover, the WNT/β-catenin signaling regulates the cadherin expression [26], mutations within the P-cadherin *CDH3* are responsible for hypotrichosis and other pleiotrophic effects, such as juvenile macular dystrophy [27] and split hand/foot malformation [28]. In addition to the Wnt signaling, the tumor necrosis factor (TNF) pathway, which is mainly composed by *EDA-A1*, *EDAR* and *EDARADD*, has been shown to play a crucial role in morphogenesis and development of the HF [29]. Mutation within the *EDA-A1* [30], [31], *EDAR* [32] and *EDARADD* [33], [34] genes are responsible for hypohidrotic ectodermal dysplasia (HED) characterized by abnormal development of hair, teeth, and eccrine sweat glands. A different signaling pathway controlled by *LIPH*, *LPA* and *LPAR6* plays a crucial role in hair growth in humans. The LIPH gene is known to produce 2-acyl LPA from phosphatidic acid (PA) [35] *LPA* promotes hair growth *in vivo* [36] in its receptor, *LPAR6*, is abundantly expressed in the inner root sheath of the HF and is involved in structural integrity of the hair shaft [37]. Mutations within this lipid signaling pathway are associated with hypotrichosis and woolly hair syndromes [37]–[41].

The pelage of many mammals consists of three hair types. The long and straight guard hairs form a top coat, and the thinner awn hairs and fine undulating down hairs form the undercoat or wool hair [42]. The hair shaft that emerges from the surface of the skin displays a wide variability in texture among individuals from different mammals. Many domesticated species have unique and or fixed hair phenotypes. Curly and wired hair are common traits across dog breeds, and the responsible mutations were identified within two genes, *KRT71* and *RSPO2,* respectively [6]. In rabbits, a deletion in exon 9 within *LIPH* is responsible for the rex hair phenotype [5].

In cats, coat color and pelage types are often selected as the main trait for developing a breed. The rexoid coat in the domestic cat was first described by Jude in 1953 [43] and by Searle & Jude in 1956 [42] as a wavy coat, absent of guard hairs. Six hairless or rexoid pelage mutations have since been documented in the domestic cat [44]–[48]. One of the newest rexoid mutations in cats, Selkirk Rex, has been recently documented [49], and two additional scientifically uncharacterized, curly coated breeds are currently recognized by cat registries; American Wirehair and LaPerm (The Cat Fanciers’ Association [CFA] http://www.cfa.org/client/home.aspx and The International Cat Association [TICA] http://www.tica.org/index.php) and others may be in development. Gandolfi *et al*. (2010) identified two recessive alleles (*re* and *hr*) within *KRT71*, a crucial gene for keratinization in the hair follicle. These alleles are responsible for curly - rexoid hair and hypotrichosis in the curly coated Devon Rex, one of the oldest rexoid mutations, and the hairless Sphynx breed, respectively [4].

**Figure 1:**
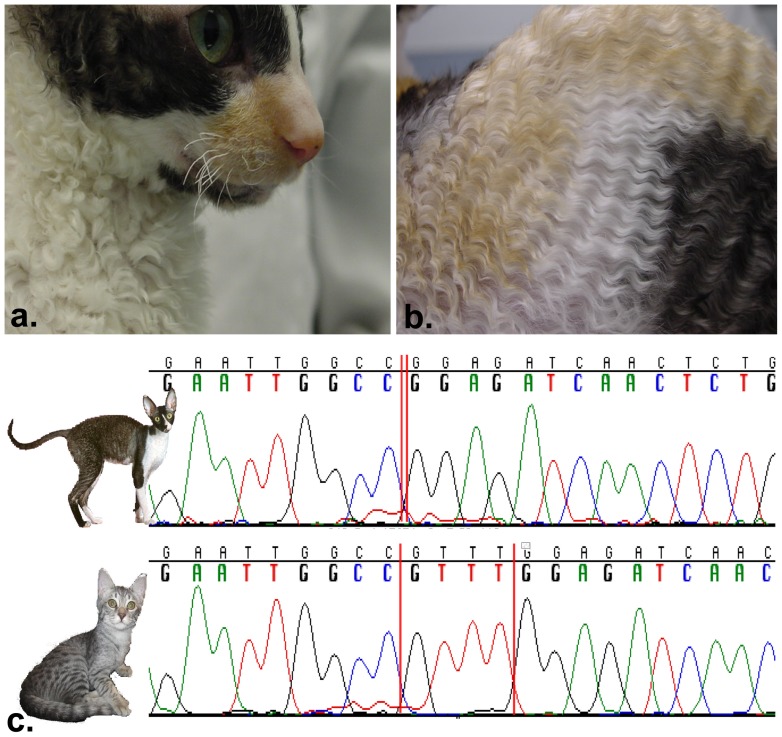
Phenotype and *P2RY5* mutation in the Cornish Rex cat. a) An adult Cornish Rex with broken whiskers. b) The curly coat presentation of “Marcel” waves. c) Electropherogram representing the four bases deletion within *P2RY5* exon 5, c.250_253delTTTG, in a Cornish Rex (top) and a control domestic shorthair (bottom).

One well established rexoid breed is the Cornish Rex, which originated from a litter of barn cats in Cornwall, England in 1950 [42], [50]. Their coat falls in washboard-like waves, also known as Marcel Waves (Figure 1a,b). The hair is very short, lies close to the body and is very soft to the touch due to the lack of guard and various awn hairs [42], [43]. Searle & Jude (1956) [42] described a second rexoid cat, the German Rex which also has a short and curly coat. The majority of Cornish and German Rexes have bent and twisted vibrissae (Figure 1a). The coat variant is inherited as an autosomal recessive monogenic trait and previous breeding studies indicated that these two Rex mutants are either identical or are different alleles at the same locus [45]. Breeding studies with Devon Rex has demonstrated that these two phenotypes are non-allelic as well [45]. Extensive artificial selection was applied to the Cornish Rex *de novo* mutation, causing fixation in the breed. Modern cat breeds have been established over the past 150 years through intense artificial selection for generally aesthetic traits caused mainly by simple single-gene variants. Many of these traits were present in random bred cat populations, pre-breed development, segregating under neutral selection or with milder positive or negative selection due to human and environmental influences. For example, *Dilute* (*melanophilin - MLPH*) is a common color variation in domestic cats [51], found in many breeds and populations around the world, however, fixed and a breed defining mutation for the Russian Blue, Korat and Chartreux. Aesthetically pleasant *de novo* mutations in cats often undergo rapid and intense positive selection to establish new breeds, which often reach fixation, or almost, within the breed population. This fixation complicates classical genome mapping studies attempting to localize the trait since controls, cats without the trait, are not available in the same population. Recent advances in feline genomics, including a 3× reference sequence [52], [53], the construction of a radiation hybrid map [54] and the development of the SNP genotyping array have enable systematic genome-scan studies [55]. By applying the statistical method *d_i_*, that measures the locus specific divergence in allele frequencies for each breed on unbiased estimates of pair-wise *F_st_*, as well as known measures such as Tajima’s D and nucleotide diversity, dominant or recessive breed-specific mutations can be detected [56], [57]. Loci under artificial selection and responsible for racing aptitude, type of gait and size in horses were recently identified using the *d_i_* statistic [57].

To localize the gene responsible for the Cornish Rex rexoid hair coat, data from the validation of the Infinium Feline 63K iSelect DNA array was analyzed by several methods, particularly an analysis for signatures of selection using *d_i_*, which measures the level of population differentiation and lineage specific recent or pre-existing selective events [56]. A region with a strong signature of recent artificial selection was identified and supported by a block of homozygosity, which led to the mutation analysis of candidate genes responsible for the hair texture diversification within the span.

## Results

### Clinical Presentation, Histology, and Ultrastructure

Cornish Rex cats in the study had the breed typical distinctive short and soft hair coat with “Marcel” wave over the entire body, legs and tail (Figure 1a, b). The very short hair on the face was not curled. The vibrissae (whiskers) were curly and were often broken and short (Figure 1b). The hair was easily plucked from the curly coated Cornish Rex and showed irregular winding on visual inspection.

Histologically, the skin contained numerous primary and secondary hair follicles, each of which contained a hair shaft. The keratin layer of the follicles often lacked the normal fibrillar appearance and was instead more globular and homogeneous. This change was seen in both primary and secondary hair follicles, but more prevalent in primary hair follicles. The Cornish Rex cats lacked thick hairs and hair shaft diameters ranged from 12 - 24 microns while the non-curly cat hairs ranged from 7 - 75 microns in diameter.

### Locus-specific Divergence and Signatures of Selective Sweep

**Figure 2:**
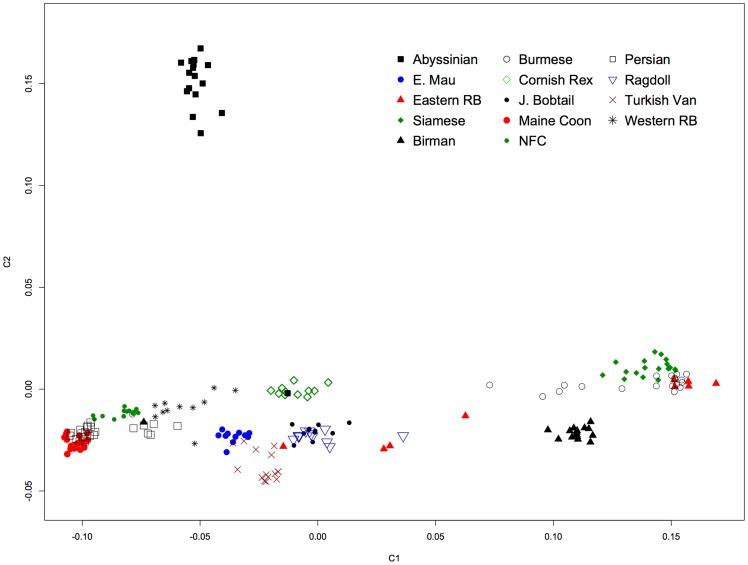
Multi-dimensional scaling of cat breeds and populations for Cornish Rex selective sweep analysis. Multi-dimensional scaling of the twelve breeds and two random bred populations.

**Figure 3:**
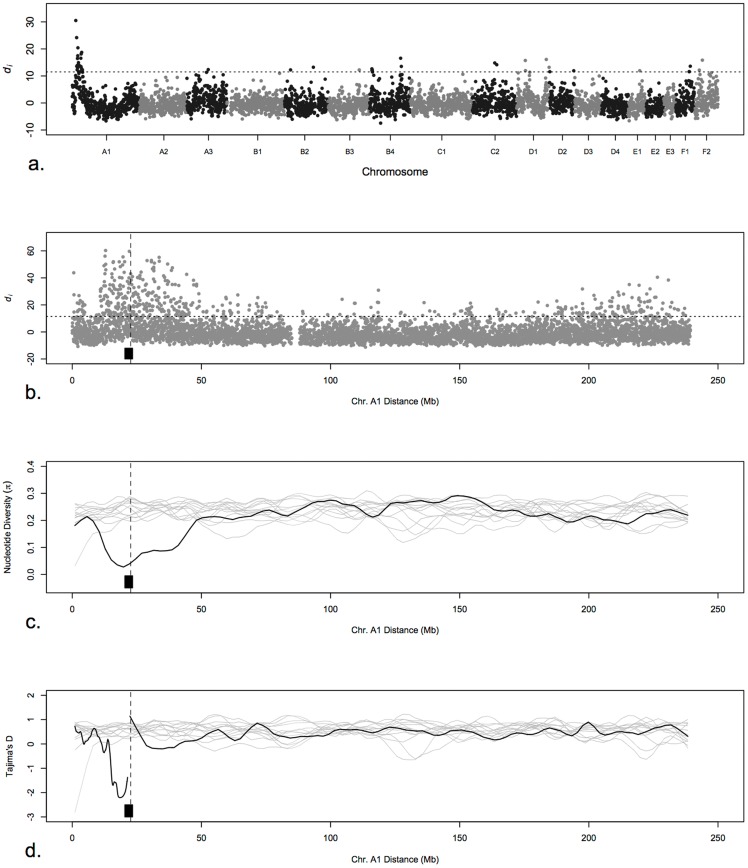
Signatures of selective sweep on chromosome A1 in Cornish Rex breed. a) Genome-wide *d_i_* values, displaying significance of chromosome A1 in Cornish Rex tested against all other cat populations. The *d_i_* value is plotted on the y axis and each autosome is shown in the X axis in alternating colors. Each dot represents one 500 Kb window. The dashed horizontal line represents the 99^th^ percentile of the empirical distribution of *d_i_*. b) *d_i_* values for each SNP on chromosome A1. c) A scan of Tajima’s D estimate along chromosome A1. Black line corresponds to Cornish Rex breed and gray lines correspond to other populations. d) A scan of nucleotide diversity along chromosome A1. Black line corresponds to Cornish Rex breed and gray lines correspond to other populations. Black block represents the 3 Mb homozygosity block detected in Cornish Rex and the dashed vertical line represents the mutation location in *P2RY5*.

Eleven cat breeds and two random bred populations of cats were compared to the Cornish Rex breed to determine signatures of selection that would identify the curly coat mutation that is fixed and unique to this breed. To visualize the relationship of the cat breeds and populations used in the analysis, the initial dataset of 247 samples was assessed for population structure by multi-dimensional scaling (MDS). The cat array has 62,897 SNPs (supporting information 63Ksnp_list); the MDS was performed on 56,811 SNPs after missingness (>0.1) and minor allele frequency SNPs (<0.05) removal. The first two dimensions were plotted as Figure 2. After removing the X chromosome and unknown chromosome SNPs, 54,044 SNPs were included in the *d_i_* statistic, which is a function of pair-wise *F_st_* between the Cornish Rex breed and the remaining breeds/populations [56]. The *d_i_* statistic was calculated only for the autosomal SNPs in non-overlapping 500 Kb windows, with a number of SNPs per window of at least 4. The average value of *d_i_* was plotted for each window (Figure 3), 53,916 SNPs were evaluated within 4,591 windows (99 SNPs were excluded as part of outlier windows), averaging 12.1 SNPs per window. Forty-four windows were in the upper 99^th^ percentile of the indicative distribution, suggesting signatures of selection (Table S1 in File S2). The maximum *d_i_* value (30.51) occurred for a window on chromosome A1, from position 12,525,126 to position 12,981,072 (SNPs positions can be found in the supporting information file 63KsnpR.map). Two additional windows with high *d_i_* values were also found on chromosome A1 at starting positions 15,558,518 and 20,493,322, respectively. Individual SNPs on chromosome A1 at positions 12,900,880, 22,091,468 and 12,679,880 showed the highest SNP *d_i_* value (Table S2 in File S2).

In addition, a genome-wide scan of signatures of selective sweeps was performed by estimating the values of Tajima’s D and nucleotide diversity over an overlapping sliding window along each chromosome for Cornish Rex and 13 control populations. Genome-wide signatures of selective sweep exclusive of Cornish Rex and represented by negative values of Tajima’s D were identified on chromosome A1 (Figure S1 in File S1). Similarly, a region of reduced nucleotide diversity was present in chromosome A1 (Figure S2 in File S1). The region on chromosome A1 exhibited both a negative value of Tajima’s D and reduced nucleotide diversity (Figure 3). None of the other populations showed any signs of selective sweep in these regions identified in the Cornish Rex.

### Homozygosity Analysis

**Figure 4:**
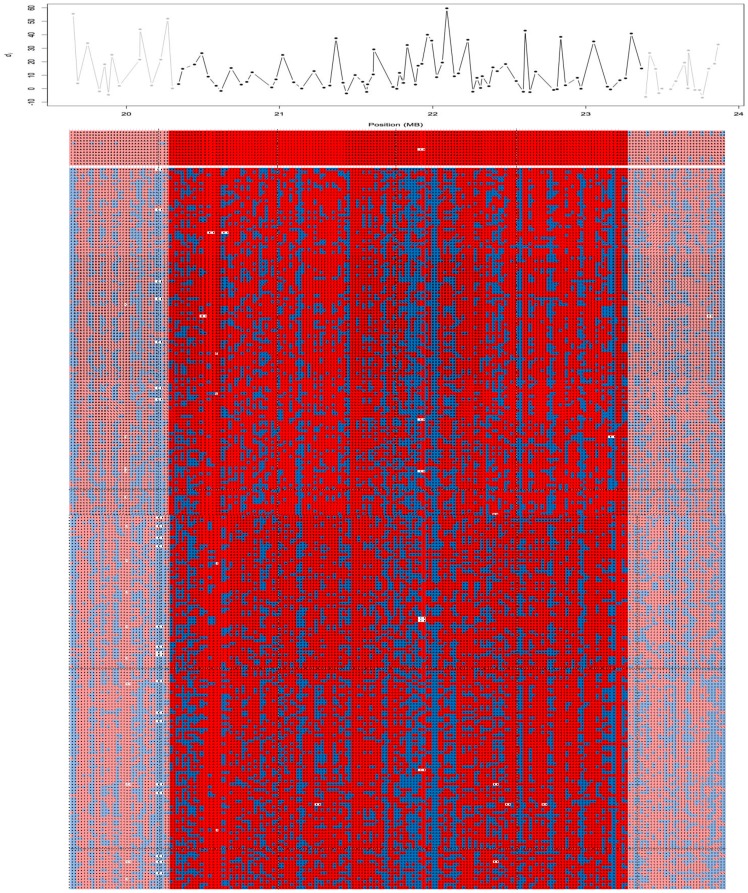
A SNP based *d_i_* in the chromosome A1 region and haplotype of Cornish Rex and control populations. The upper graph shows the *d_i_* value for each SNP in the region within the 3 Mb shared haplotype across all Cornish Rex cats. Below, each SNP is represented by two alleles in two contiguous boxes. Boxes colored in red represent the major allele of Cornish Rex, while the blue color represent the minor allele. Faded color starts at the end of each boundaries of the haplotype block in the Cornish Rex samples. The first 12 lines of boxes represent the haplotype of each Cornish Rex included in the analysis, while below are all the other samples included in the analysis.

Homozygosity analysis was conducted on all the available Cornish Rex samples (n = 12). Excluding the blocks detected on the X chromosome, three homozygous blocks were detected (Table S3 in File S2) across all samples. The first two homozygous blocks were on chromosome A1 in close proximity to one another and consisted of ∼300 Kb (8 SNPs, from position 18340220 to 18636486) and ∼3.1 Mb (74 SNPs, from position 20301116 to 23456462), respectively. The second block was detected on chromosome B4 and spanned ∼1 Mb (29 SNPs, from position 7032654 to 8047404). No homozygous blocks were identified for the other populations in these locations (data not shown). A unique single haplotype across over 3 Mb, spanning positions 20,341,274 to position 23,364,238, was identified (Figure 4). The haplotype consisted of 74 SNPs. The position of the homozygous blocks overlapped with the areas of selective sweep (Figure 3, Figure 4). Inspection of genes within the two blocks on chromosome A1 revealed the presence of 31 genes annotated in humans, with several functions as listed in Table S4 in File S2. Within the 3 Mb block on chromosome A1, a strong candidate gene involved in the maintenance of hair growth and texture, *LPAR6*, was selected for further analysis. The block on chromosome B4 contained two genes, but no candidate genes were recognized. Detailed haplotype analysis on the chromosome B4 region revealed that the block consisted of 13 contiguous SNPs from position 7,341,020 to position 7,757,024 (data not shown).

### LPAR6 Analysis and Genotyping

The coding sequence (CDS) for *LPAR6* (GenBank accession no. JN977053) and 3′ UTR were sequenced in 21 cats representing all the rexoid and hairless variants of cat breeds (Table S5 in File S2). The *LPAR6* CDS is 1,035 bp, coding for 344 amino acids in the domestic cat. Except for four identified variants, all cats were conserved across the entire CDS. Comparison of DNA sequence from all the cats revealed that the Cornish Rex has a 4 bp deletion in exon 5, c.250_253_delTTTG, which causes a frame shift and premature termination codon at position 92 of the protein (Figure 1c). The mutation within *LPAR6* leads to a protein product truncated prior to the third trans-membrane domain. Three additional sequence variants were identified, including two mutations in the 5′ UTR of the gene (c.-194G>A, c.-72C>T) and one synonymous variant (c.63C>T). Straight coated cats had the c.-72C>T and the c.63C>T mutations suggesting these mutations do not affect coat phenotype. To verify tissue specific expression, the deletion was typed on hair root bulb mRNA derived from a Cornish Rex and control cat and on the Cornish Rex skin biopsy. The deletion was confirmed in the Cornish Rex and the control showed the wild-type RNA transcript (Figure 1c, Figure S3 in File S1).

Thirty-nine unrelated Cornish Rex cats and 51 additional cats from other rexoid, hairless and random bred breeds and populations were genotyped for the deletion (Table S6 in File S2). All Cornish Rex were homozygous for the deletion. The same deletion was detected in a second breed, the German Rex, in a homozygous state in two curly coated cats and heterozygous in two straight haired cats. The two heterozygous cats were tested for the *KRT71^re^* mutation but the Rex (*R*) allele was not detected (data not shown). All the random bred cats and the other rexoids had wild-type genotypes, suggesting that the identified mutation is causative only for the curly coated rexoid phenotype in Cornish and German Rex cats.

## Discussion

In this study, a single gene, recessive mutation that reached fixation in the Cornish Rex within the past 75 years, with complete penetrance and consistent and clearly defined presentation, was evaluated. The mutation is fixed within Cornish Rex, defining the breed, and is only present and segregating in another cat breed called German Rex. No within population control cats are available to perform a classical genome-wide association study. Consequently, using the Illumina Infinium Feline 63K iSelect DNA array validation data, the identification of regions under selection near fixation within the breed (*d_i_*) was used to suggest the chromosomal region harboring the breed defining trait.

Although linkage disequilibrium (LD) among cat breeds extends from 20 - 400 Kb and LD in Cornish Rex is ∼60 Kb [58], the nature of the mutation under selection is likely to cause a local amplified extent of LD, thus 500 Kb non overlapping windows were chosen for the calculation of the *d_i_* statistic. Many putative windows indicative of selection were identified within the Cornish Rex breed, likely due to the low number of individuals used in the study. The significance of the region on chromosome A1 was confirmed by nearly 50% of significant windows found on this chromosome, suggesting a strong artificial selection on the phenotypic trait characteristic of the breed and supporting the relatively recent fixation of the mutation in the breed. Moreover, this signature of selection was supported by homozygosity.

The homozygosity analysis identified two regions in the genome where all the Cornish Rex shared an extended span of homozygosity. The largest region is on chromosome A1 that contains 31 coding genes and harbors the gene *LPAR6*, which represented a strong candidate gene. A second shorter homozygous block was identified on chromosome B4, which includes two genes, neither an obvious candidate gene controlling hair morphology or development. Haplotype analysis for chromosome B4 also failed to identify a unique haplotype block shared across all the samples, suggesting the region is under positive selection, although no obvious role of the genes in the area was suggested. These same regions were confirmed by a reduction in nucleotide diversity, which also constitutes a signature of selective sweep. Genome-wide, only the region on chromosome A1 was suggested by Tajima’s D values, which connotes positive selection. The presence of other blocks in the genome, beside the one harboring the rexoid locus, was expected, since the Cornish Rex have other unique phenotypic features. The *White Spotting* coat coloration [59], [60] is, in fact, highly prevalent within the breed, and the Cornish Rex also exhibit distinctive morphology, including a distinctive head, ear shape, and body type. Uninvestigated regions may also contain important information regarding polymorphisms implicated in the determination of significant traits within the Cornish Rex breed and require further examination.

The regions identified by the selective sweep and homozygosity mapping methods were refined and prioritized by phasing the haplotype in the region of interest. The locus on chromosome A1 was fixed for an extended haplotype of over 3 Mb (74 SNPs), allowing for detection via *d_i_* measures in non overlapping windows, and suggesting extreme and continued selective pressure for the haplotype that is driving this signature of selection. The haplotype was examined for candidate genes controlling hair morphology and development and a feasible candidate gene for Cornish Rex curl, *LPAR6,* was identified.

*LPAR6* has 2 isoforms in humans of 2428 bp and 2911 bp, respectively. Both isoforms have a 1035 bp CDS, coding for a 344 amino acid protein [61]. The gene has five exons and the translation starts in exon 5 at position 1144 of the transcript. The human *LPAR6* gene contains four potential extracellular domains, four cytoplasmic domains and seven predicted hydrophobic trans-membrane regions (http://au.expasy.org/uniprot/P43657). The receptor is abundantly expressed in the inner root sheath of the human hair follicle [37] as well as in placenta, thymus, spleen, prostate and epidermis. The sequence of *LPAR6* is highly conserved in vertebrate evolution and has homologues in zebrafish, also confirmed by a 92.7% homology between cat and human.

The Cornish Rex has a 4 bp deletion in exon 5, c.250_253_delTTTG, which causes a frame shift and premature termination codon at position 92 of the protein. The mutation within *LPAR6* leads to a protein product truncated prior to the third trans-membrane domain. Expression of the truncated *LPAR6* was supported in the Cornish Rex cats through the hair RNA. In the truncated protein of the cat, the last four trans-membrane domains are deleted, along with 3 extracellular and 2 intracellular domains, suggesting a complete loss or reduction of the receptor function. The diverse breeds of cats analyzed had minimal genetic variation within the coding sequence of *LPAR6*, having only one missense mutation. All Cornish Rex were fixed for the mutation and no straight haired cats carried the mutation. Only the German Rex breed, a breed with few individuals and developed from Cornish Rex, also had the *LPAR6* mutation. The two heterozygous German Rex had straight hair and the mutation was not allelic to some other unknown rexoid mutations. Hence, the German Rex was established from a mutation that characterized and is unique to the Cornish Rex breed, which is also confirmed by the phenotypic similarities between the two breeds, from the nature of the curls and the bent and twisted vibrissae. German Rex are not fixed for the curly mutation because the breed never became popular and the breed is still represented by a small pedigree of cats. Since the complete UTR sequence of *LPAR6* is not yet acquired, the locus cannot be excluded as the candidate for other rexoid mutations, such as LaPerm or Tennessee Rex, both breeds are undocumented rexoid mutations.

The hair follicle is a skin appendage with a highly complex structure within which the keratinization process results in the formation of the hair shaft. A crucial role in the development of the hair shaft is played by lysophosphatidic acid (*LPA*), which is an active lipid abundant in the hair follicle, serving as a protective barrier and promoting hair growth [36]. LPA is shown to serve as a ligand for the purine and pyrimidine nucleotide receptor *LPAR6* [62]–[64]. In humans, *LPAR6* is located on chromosome 13q14 and missense mutations [38], [65]–[67], insertions [37], [62] and deletions [37], [62], [68], [69], within the gene were reported as responsible for autosomal recessive woolly hair in Pakistani, Turkish and Indian families, respectively. Dominant or recessive mutations in extremely conserved regions within *LPAR6* can cause tightly curled or wooly hair [70] and/or hypotrichosis in human [38], [65]–[67]. Cornish Rex presents a minimal degree of hypotrichosis and tightly curled woolly hair. The term hypotrichosis refers to a group of hereditary alopecia characterized by diffuse and progressive hair loss [68], that can be localized and characterized by fragile hairs, that break easily, leaving short and sparse hair. Woolly hair (WH) refers to a group of hair shaft disorders that are characterized by fine and tightly curled hair. WH can be autosomal recessive (ARWH) or autosomal dominant (ADWH) and can manifest as a syndromic or non syndromic form. In its syndromic form, the hair shows some structural anomalies, including trichorrhexis nodosa and tapered ends [71]. Hypotrichosis can be an associated feature of ARWH. *LIPH,* together with *LPAR6,* define a developmental axis of hair growth and shape and hypotrichosis is speculated to be associated with woolly hair when the pathological findings and structural abnormalities in the hair follicle are severe. Although the alteration of the Cornish Rex *LPAR6* protein is severe, the breed presents mild hypotrichosis compared to human, where point mutations affecting the binging of LPA with the receptor can be associated with complete hair loss [65]. The role of *LPAR6* protein in the cat hair follicle has not been determined, but potentially, either the different genetic background as compared to humans or the compromised residual activity of the Cornish Rex protein in the root sheath causes a compromised function in anchoring the hair shaft, which leads to the WH phenotype.

Several causative genes containing mutations for hereditary hair diseases have been identified in the recent years, but this is the first characterized mutation within *LPAR6* associated with a curly phenotype in an animal other than humans. The characterization of these mutants demonstrates the importance of *LPAR6* in the maintenance of the structural integrity of the hair shaft and helps elucidate information about the biology of the hair follicle. Despite these advances, additional genes whose mutations underlie hereditary hair disease remain to be identified and the domestic cat could be used as a model for the other human and animal hair diseases that are not yet linked to identified genes.

## Materials and Methods

### Clinical Description and Sampling

Private owners of Cornish Rex cats were recruited to voluntarily participate in the study. Samples collection and use of cats for the control samples was approved under IACUC animal protocols 16691 and 15933. Cats were examined at the University of California – Davis, William R. Pritchard Veterinary Medicine Teaching Hospital. Skin biopsy samples were obtained from two privately owned cats after acquiring informed owner consent. Cats received a dermatologic examination. An area along the dorsal cervical region was selected and a subcutaneous injection of 0.5 ml of lidocaine was administered to provide local analgesia to permit collection of the skin biopsy sample. Skin samples were obtained using an 8 mm skin biopsy punch. The biopsy site was closed using 3-0 nylon suture. The skin biopsy was bisected: one portion was placed in 4% formalin and embedded in paraffin and a second portion was transferred into RNA later (Qiagen, Valencia, CA). Hairs were sampled using a hemostat from random locations over the dorsum and scapular region. Hair bulbs were stored in RNAlater and frozen at −80°C (Qiagen).

### Samples Collection

As part of the preliminary testing of the Infinium Feline 63K iSelect DNA array (Illumina, Inc., San Diego, CA), DNA for the Cornish Rex and control straight haired cats from twelve genetically distinct breeds and two random bred populations was isolated from blood, tissue or buccal swab samples from 247 cats. The twelve breeds included Abyssinian, Maine Coon, Persian, Norwegian Forest Cat, Egyptian Mau, Japanese Bobtail, Ragdoll, Turkish Van, Cornish Rex, Burmese, Birman and Siamese. Two random bred populations, with Eastern and Western origins (mean population sample size = 17.64) (Table S8 in File S2). Most cats were selected to not share parents or grand-parents. DNA was isolated using the DNAeasy Kit (Qiagen) and concentrated using Genomic DNA clean & Concentrator kits (Zymo Research, Irvine, CA) when necessary. Quality and quantity of DNA was confirmed by visualization with gel electrophoresis and by optical density using the BioPhotometer (Eppendorf, Hamburg, Germany). Approximately 20 µl of DNA at >30 ng/µl was submitted for testing. Array assays were performed by Illumina, Inc. (San Diego, CA). Populations structure was performed with a classic multi-dimensional scaling (MDS) with 2 dimensions after SNPs removal for missingness (0.1) and minor allele frequency (0.05) in PLINK [72].

### d_i_ Statistic

Cornish Rex locus specific divergence (*d_i_*) was calculated as presented by Akey *et al* [56] on the available 11 breed and 2 random bred cat populations. SNPs assigned to the unknown chromosome and X chromosome, or with a MAF <0.05 were removed from the analysis. Pair-wise *F_st_* was calculated using the function (*Fst*) in the R package, *pegas* [73] for each SNP between the Cornish Rex population and each of the other 13 populations. Subsequent calculations of *d_i_* values were performed using scripts written in R using the following procedure. For each SNP, *F_st_* value between Cornish and each of the 13 populations is calculated as follow (*F_st_* (at SNP1 between Cornish Rex and population 1) – mean (*F_st_* across all markers between Cornish Rex and population 1) divided by the standard deviation of *F_st_* (across all markers). This quantity is calculated for each of the 13 population against Cornish Rex, independently. At each marker, the *d_i_* represents the sum of the quantities calculated above. Thus, the *d_i_* is the sum of 13 quantities that correspond to the *F_st_* values between Cornish and each of the 13 populations.

Non-overlapping 500 Kb windows containing at least four SNPs were considered and *d_i_* values within the interval were averaged as previously implemented [56], [57]. The 99^th^ percentile threshold was used to determine significance of the obtained *d_i_* values.

### Genome-wide Scans

Genome-wide scans of Tajima’s D and nucleotide diversity were conducted in R using *pegas* package [73]. Tajima’s D and nucleotide diversity was calculated for an overlapping sliding window of 50 SNPs. The position of the median SNP was used to correspond to window’s value of the statistic. Each statistic was plotted as a function of distance/position along each chromosome. The resulted line was smoothed using the function *lowess* in R [74].

### Region of Homozygosity Analysis (ROH)

Homozygosity analysis was conducted using PLINK [72]. A window of 25 SNPs (∼1000 Kb) was surveyed for homozygosity, allowing five missing genotypes and a single heterozygous. A homozygous block was defined by five SNPs (or ∼250 Kb) and the threshold of homozygosity match was selected as 0.99. The consensus homozygosity block was defined as the overlapping homozygosity block from each individual using the command (–*homo-group*). Linkage disequilibrium (LD) was investigated, exporting SNPs from position 18,000,000 to position 25,000,000 on chromosome A1 (n = 179 SNPs), and visually inspected.

### LAPR6 Genomic and mRNA Analysis

The genomic analysis of *LPAR6* was conducted on genomic DNA from 21 cats (Table S1 in File S2) including eight rexoid breeds, three hairless breeds and three shorthair cats. The tissue samples stored in RNA later (Qiagen) were used to isolate RNA using PureLink RNA mini Kit (Invitrogen, Carlsbad, CA).

The complete CDS of *P2RY5* is publicly available and can be found on chromosome A1∶226430008-22644033 (www.ensambl.org). *LPAR6* has 5 exons, the first four are not translated, and the start codon is within exon 5. In this study, the full CDS, 3′ UTR and the 5′ UTR that encompassed only exon 5, was analyzed in 21 cats representing all the well known rexoid and hairless variants (Table S1 in File S2). Primers were designed using Primer3plus (http://www.bioinformatics.nl/cgi-bin/primer3plus/primer3plus.cgi) in the UTR regions, flanking the exons containing the full CDS. Primers were tested for efficient product amplification on a DNA Engine Gradient Cycler (MJ Research, GMI, Ramsey, MN). Primer sequences and amplicon size for each primer pair are shown in Table S7 in File S2. PCR and thermocycling conditions were conducted as previously described at 2.0 mM Mg^2+^ at 62°C [55]. The PCR for genotyping used 1.75 mM Mg^2+^. The PCR products were purified with the ExoSap (USB, Cleveland, OH) per the manufacturer’s recommendations and directly sequenced using the BigDye terminator Sequencing Kit v3.1 (Applied Biosystem, Carlsbad, CA). The sequencing products were purified with Illustra Sephadex G-50 (GE Healtcare, Piscataway, NJ) according to the manufacturer’s recommendations, and electrophoretically separated on an ABI 3730 DNA analyzer (Applied Biosystems). Sequences were verified and aligned using the software sequencer version 4.9.1 (Gene Codes Corp., Ann Arbor, MI). Complementary DNA templates were synthesized by reverse transcription of 1 µg of mRNA from hair bulbs of a control cat and the Cornish Rex cat with the PolydT primer to obtain the 3′UTR of *P2RY5* and partial 5′ UTR. Primers for the cDNA analysis are provided in Table S7 in File S2.

### LPAR6 Mutation Genotyping

To confirm the identified mutation as causative, an additional set of samples consisting of 39 cats from the Cornish Rex breed, and 43 cats from nine breeds representing multiple coat phenotypes, as well as eight random bred cats, were used for genotyping (Table S6 in File S2). A PCR reaction using the P2RY5-Fdel with a fluorescence label and P2RY5-Rdel (Table S7 in File S2) was performed and electrophoretically separated on an ABI DNA analyzer (Applied Biosystems). The predicted size of the wildtype allele was 372 bp and 368 bp for the mutant and verified using the software STRand [75]. PCR to evaluate the presence of the *KRT71^re^* allele in the two heterozygous German Rex was conducted as described in Gandolfi *et al*. 2010.

### Histological and Ultrastructure Evaluation of Hair Follicles and Hair Shafts

Morphological features of hair follicles and hair shafts were evaluated on 5 µm paraffin sections routinely stained with hematoxylin and eosin. For hair shaft ultrastructure examination, the proximal 0.5–0.8 cm of the hair shafts was first cleaned by a chloroform sonication for 5 min. The chloroform was removed and hairs dried overnight under vacuum with a chemical desiccant. Eighteen randomly selected hairs per cat were adhered to metal scanning electron microscopy (SEM) specimen mounts using dual-sided conductive carbon tape and then sputter coated with 2 nm of chromium to prevent buildup of electrostatic charge during imaging. A S-4800 field emission SEM (Hitachi, Pleasanton, CA) was operated at 0.5–2 kV accelerating voltage to generate images of the proximal 0.3–0.5 cm of each hair at 500×, 1000×, and 2000× magnification. The hair diameters in the resulting images were measured using a freehand measurement tool of the ImageJ (NIH, version 1.36b) software.

## Supporting Information

File S1 **Supporting Figures.** Figure S1. Genome-wide scan of Tajima’s D estimate in cat populations. Black line corresponds to Cornish Rex breed and gray lines correspond to other populations. A clear decrease in the values of Tajima’s D is detected in chromosome A1 in the Cornish Rex breed. The reduction in Tajima’s D values is unique to the Cornish Rex breed and supports the presence of a signature of selection. Figure S2. Genome-wide scan of nucleotide diversity in cat populations. Black line corresponds to Cornish Rex breed and gray lines correspond to other populations. A reduction in nucleoride diversity in the Cornish Rex breed is detected in chromosome A1 and is unique to the breed. Figure S3. Protein sequence aligments of *LPAR6* in different species. *LPAR6* is highly conserved in human, alpaca, chimpanzee, elephant, hedgehog, rabbit, zebrafish, cat wild-type and Cornish Rex. The Cornish Rex mutation causes a frameshift with a premature stop codon occurring at amino acid position number 92. The portion of the Cornish Rex allele that is altered relative to the wildtype is presented in bold.
(DOC)

File S2 **Supporting tables.** Table S1. SNPs with *d_i_* values above the 99^th^ percentile in the Cornish Rex analysis. Table S2. SNP coordinates in the cat genome with highest *d_i_* values for Cornish Rex phenotype. Table S3. Consensus details homozygous regions across all Cornish Rex cats. Table S4. Gene symbols and names within the 3 Mb haplotype on chromosome A1. Table S5. SNP identification of *LPAR6* in the domestic cats. Table S6. *LPAR6* genotyping results in cat breeds with pelage mutations. Table S7. PCR primers for the analysis of *LPAR6* in the domestic cat. Table S8. Breeds and populations included in the signatures of selective sweep analyses (d_i_ and homozygous regions detection).
(DOC)
