# Supplementary material for: To the Root of the Curl: A Signature of a Recent Selective Sweep Identifies a Mutation That Defines the Cornish Rex Cat Breed
Source: PLoS One. 2013 Jun 27;8(6):e67105. doi: 10.1371/journal.pone.0067105 (PMC3694948; doi:10.1371/journal.pone.0067105)
Supplement: File S1 — Supporting Figures. Figure S1. Genome-wide scan of Tajima’s D estimate in cat populations. Black line corresponds to Cornish Rex breed and gray lines correspond to other populations. A clear decrease in the values of Tajima’s D is detected in chromosome A1 in the Cornish Rex breed. The reduction in Tajima’s D values is unique to the Cornish Rex breed and supports the presence of a signature of selection. Figure S2. Genome-wide scan of nucleotide diversity in cat populations. Black line corresponds to Cornish Rex breed and gray lines correspond to other populations. A reduction in nucleoride diversity in the Cornish Rex breed is detected in chromosome A1 and is unique to the breed. Figure S3. Protein sequence aligments of LPAR6 in different species. LPAR6 is highly conserved in human, alpaca, chimpanzee, elephant, hedgehog, rabbit, zebrafish, cat wild-type and Cornish Rex. The Cornish Rex mutation causes a frameshift with a premature stop codon occurring at amino acid position number 92. The portion of the Cornish Rex allele that is altered relative to the wildtype is presented in bold. (DOC) [file pone.0067105.s001.doc]

**Supplementary figures legend:**


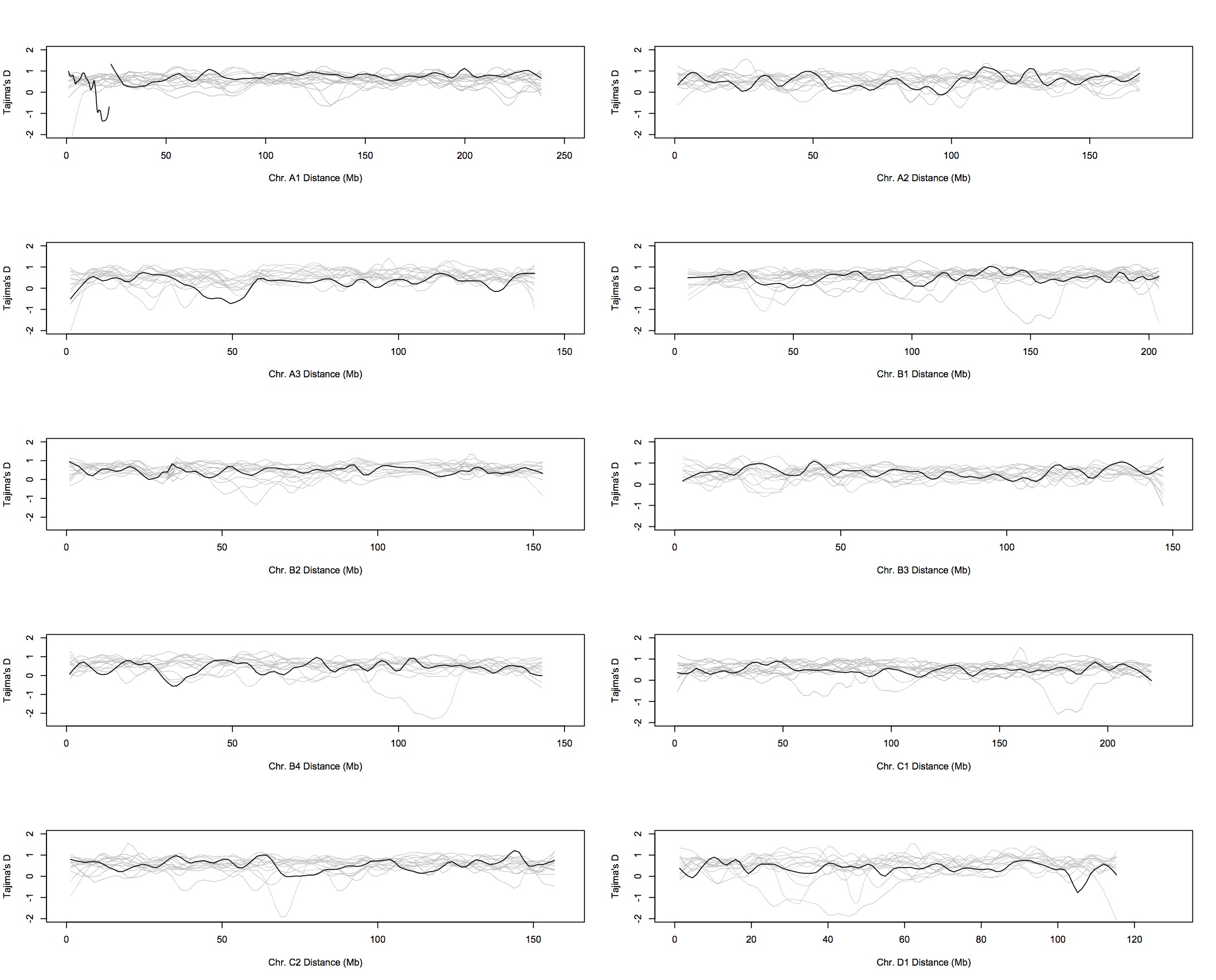


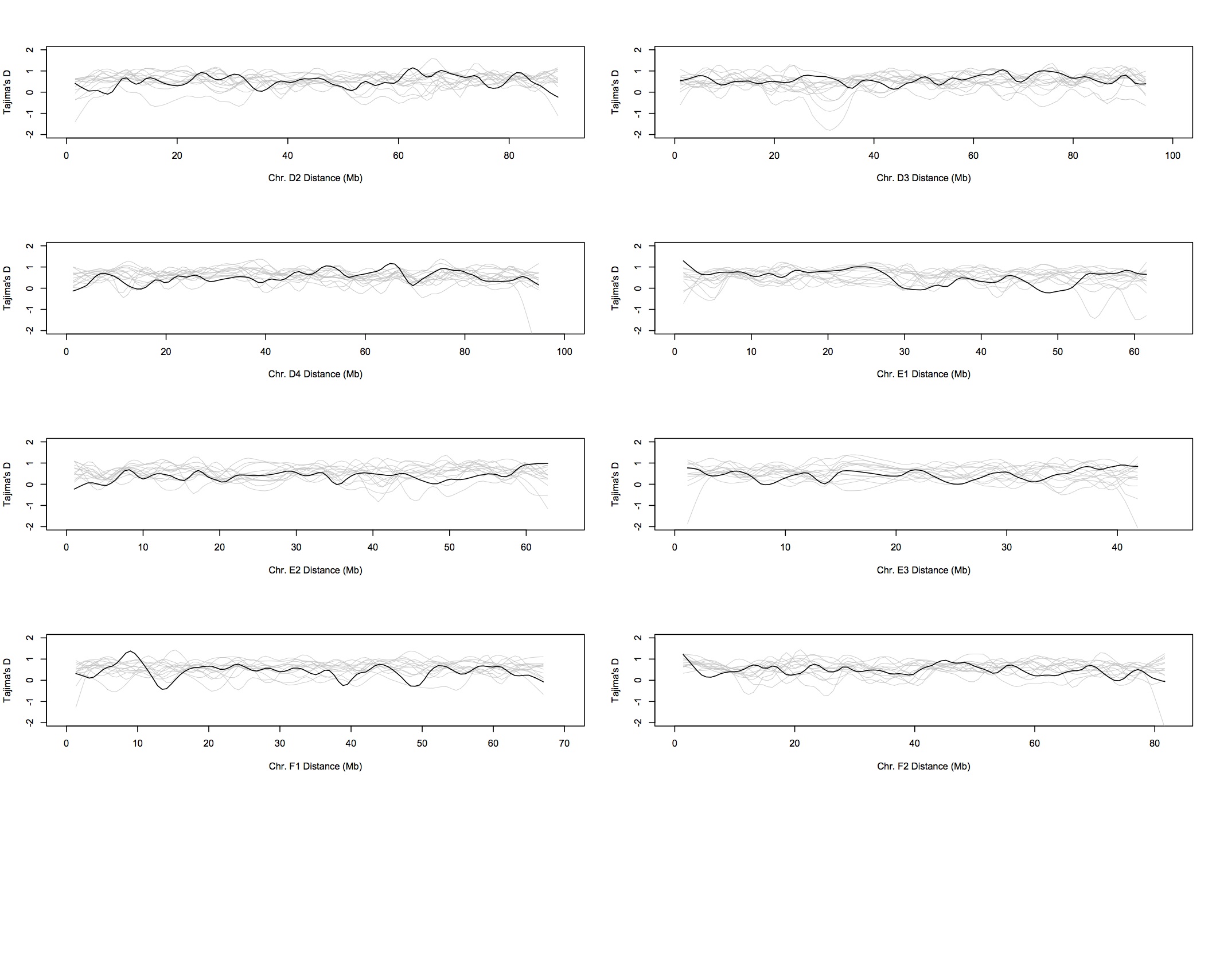


**Figure S1. Genome-wide scan of Tajima’s D estimate in cat populations.** Black line corresponds to Cornish Rex breed and gray lines correspond to other populations. A clear decrease in the values of Tajima’s D is detected in chromosome A1 in the Cornish Rex breed. The reduction in Tajima’s D values is unique to the Cornish Rex breed and supports the presence of a signature of selection.


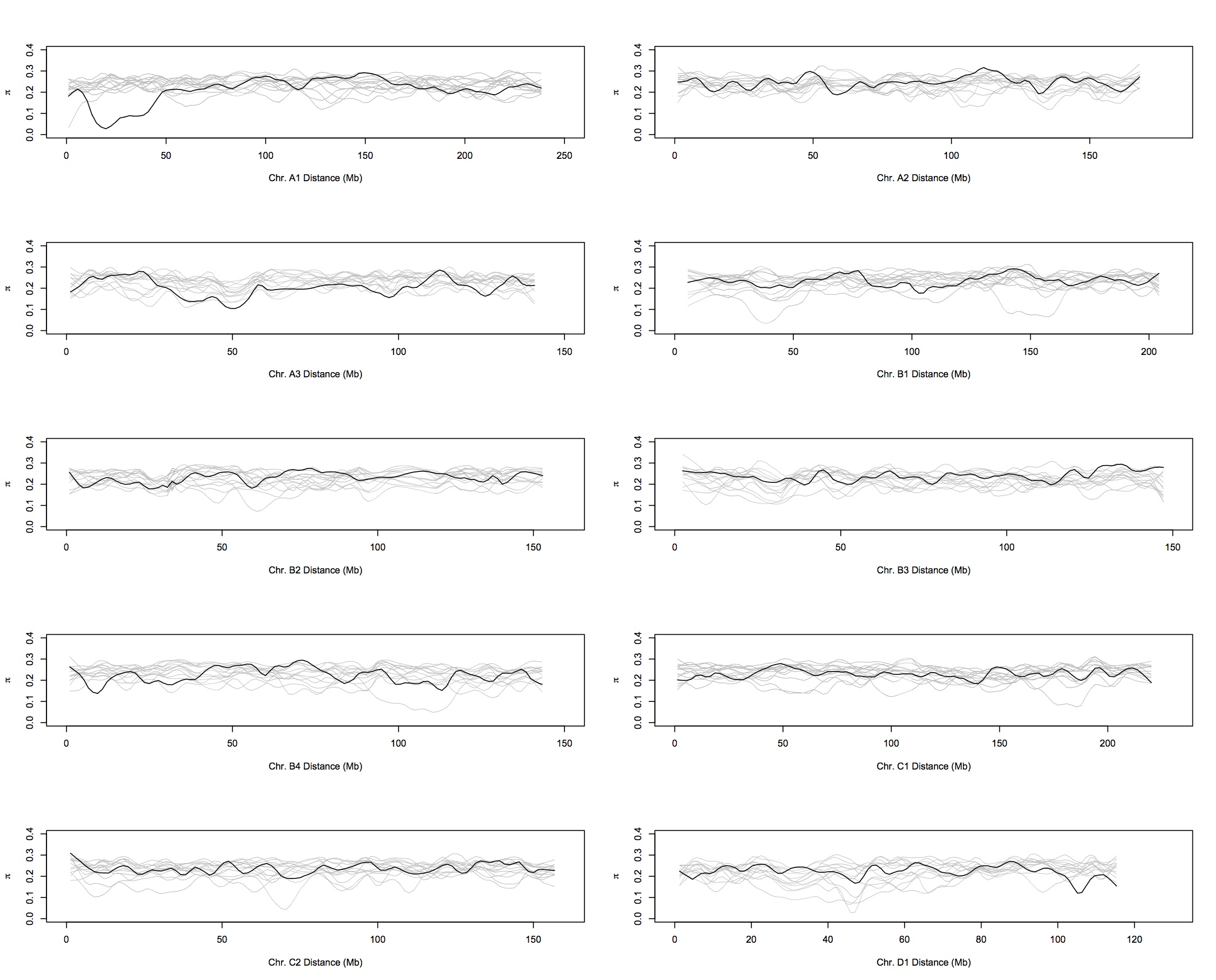


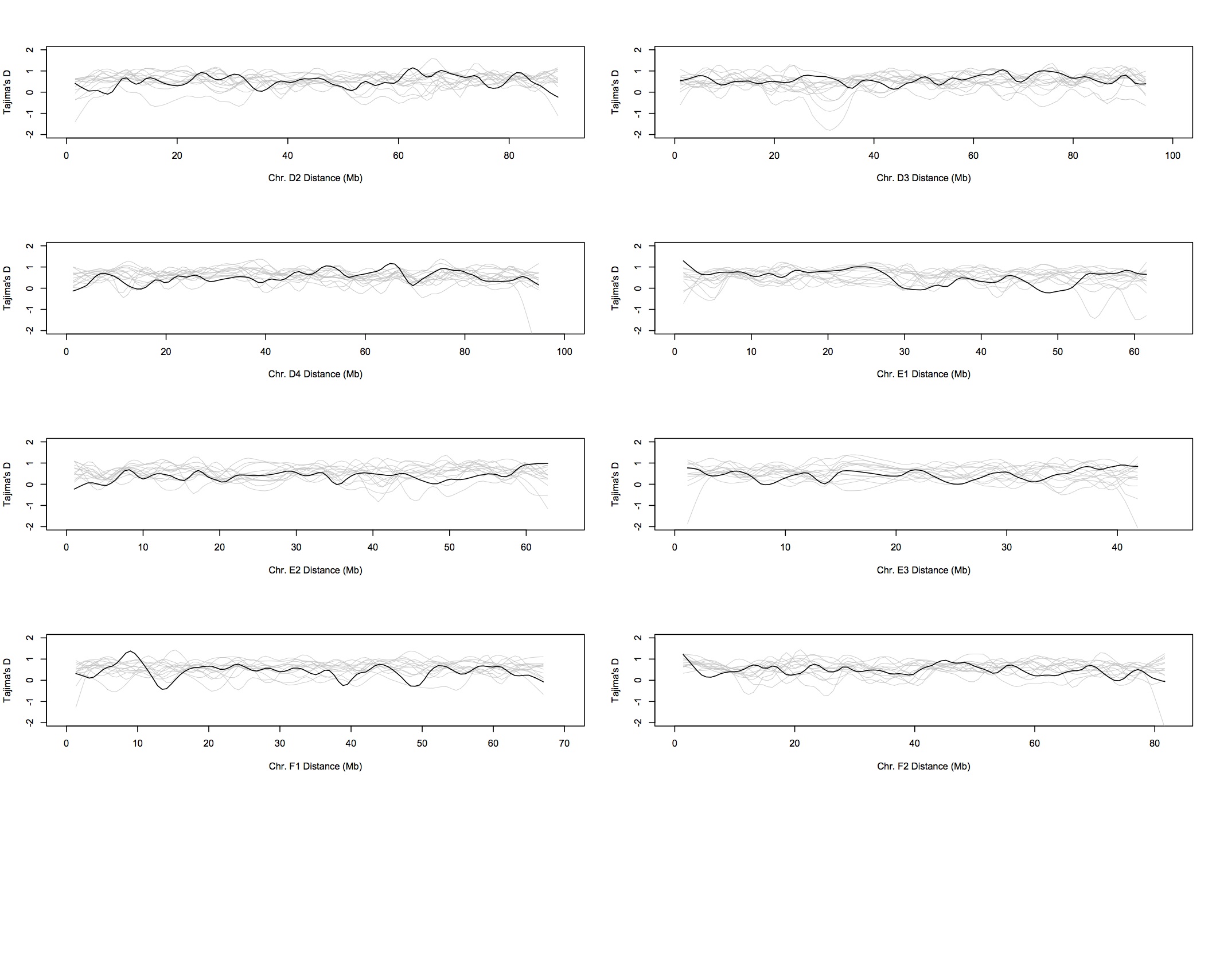


**Figure S2.** **Genome-wide scan of nucleotide diversity in cat populations.** Black line corresponds to Cornish Rex breed and gray lines correspond to other populations. A reduction in nucleoride diversity in the Cornish Rex breed is detected in chromosome A1 and is unique to the breed.

....|....| ....|....| ....|....| ....|....| ....|....| ....|....|

5 15 25 35 45 55

Human MVSVNSSHCF YNDSFKYTLY GCMFSMVFVL GLISNCVAIY IFICVLKVRN ETTTYMINLA

Alpaca MVSNNSSHCT YDDSFKYTLY GCMFSMVFVL GLISNCVAIY IFMCTLKVRN ETTTYMINLA

Chimpanzee MVSVNSSHCF YNDSFKYTLY GCMFSMVFVL GLISNCVAIY IFICVLKVRN ETTTYMINLA

Elephant MVSINSSSCS YDDSFKYTLY GCIFSMVFVL GLISNCVAIY VFICTLKIRN ETTTYMVNLA

Hedgehog MVSNNSSNC- FDDSFKYTLY GCMFSMVFVL GLISNCVAIY IFICTLKVRN ETTTYMINLA

Rabbit MVSANSSHCS YDDSFKYTLY GCMFSMVFVL GLISNCVAIY IFICTLKVRN ETTTYMINLA

Zebrafish MMTERSNCT- -NDSFKYVLY SSVFSIVFIL GLLFNMVAMY IFVCRLKMRN ETTTYMMNLV

Cat wildtype MVSNNSSQCY YDDSFKYTLY GRMFSMVFVL GLISNCVAIY IFICTLKVRN ETTTYMINLA

Cornish Rex MVSNNSSQCY YDDSFKYTLY GRMFSMVFVL GLISNCVAIY IFICTLKVRN ETTTYMINLA

....|....| ....|....| ....|....| ....|....| ....|....| ....|....|

65 75 85 95 105 115

Human MSDLLFVFTL PFRIFYFTTR NWPFGDLLCK ISVMLFYTNM YGSILFLTCI SVDRFLAIVY

Alpaca ISDLLFVFTL PFRIFYFATR NWPFGDSLCK ISVMLFYTNM YGSILFLTCI SADRFLAIVY

Chimpanzee MSDLLFVFTL PFRIFYFTTR NWPFGDLLCK ISVMLFYTNM YGSILFLTCI SVDRFLAIVY

Elephant MSDLLFVFTL PFRIFYFATR NWPFGDLLCK ISVMLFYTNM YGSILFLTCI SADRFLAILY

Hedgehog MSDLLFVFTL PFRIFYFATR NWPFGDLLCK ISVMLFYTNM YGSILFLTCI SVDRFLAIVY

Rabbit MSDLLFVFTL PFRIFYFATR NWPFGDLLCK ISVMLFYTNM YGSILFLTCI SVDRFLAIVY

Zebrafish VSDSLFVVSL PFRTFYFINR QWPFGDALCK ISVTLFYTNM YGSILFLTCI SVDRFLAIVY

Cat wildtype MSDLLFVFTL PFRIFYFATQ NWPFGDQLCK IS**VMLFYTNM YGSILFLTCI SVDRFLAIVY**

Cornish Rex MSDLLFVFTL PFRIFYFATQ NWPEINSVKF Q*-------- ---------- ----------

....|....| ....|....| ....|....| ....|....| ....|....| ....|....|

125 135 145 155 165 175

Human PFKSKTLRTK RNAKIVCTGV WLTVIGGSAP AVFVQSTHSQ GNNASEACFE NFPEATWKTY

Alpaca PFKSKTLRTK RNAKIVCIAV WLTVMGGSAP AVFLPSTHSQ GNNTSKACFE NFPEATWKTF

Chimpanzee PFKSKTLRTK RNAKIVCTGV WLTVIGGSAP AVFVQSTHSQ GNNASEACFE NFPEATWKTY

Elephant PFKSKTLRTK RNAKIVCTAV WLTVLGGSAP AVFFQSTYSK DNNTSEACFE NFPEATWKTY

Hedgehog PFKSKTLRTK RNAKIVCIAV WLTVVGGSAP AVFFQSTHSR GNNTSKACFE NFPEDTWKTY

Rabbit PFKSKTLRTK RNAKIVCIAV WLTVIGGSAP AVFFQSTHSQ GNNTSEACFE NFPEATWKTY

Zebrafish PFASRTLRTK RNAKIACGVI WVVLLSGGLT AGFVMDITSH KNET--YCFE NYSKSQWKSQ

Cat wildtype **PFKSKTLRTK RNAKIVCIAV WLTVIGGSAP AVFFQSTHSQ GNNASEACFE KFPEATWKTY**

Cornish Rex ---------- ---------- ---------- ---------- ---------- ----------

....|....| ....|....| ....|....| ....|....| ....|....| ....|....|

185 195 205 215 225 235

Human LSRIVIFIEI VGFFIPLILN VTCSSMVLKT LTKPVTLSRS -KINKTKVLK MIFVHLIIFC

Alpaca LSRIVIFIEI VGFFIPLILN VTCSSMVLRT LNKPVTLSRS -KINKTKVLK MIFVHLVIFC

Chimpanzee LSRIVIFIEI VGFFIPLILN VTCSSMVLKT LTKPVTLSRS -KINKTKVLK MIFVHLIIFC

Elephant LSRIVIFIEI VGFFIPLILN VTCSSMVLRT LNKPVTLSRS -KINKTKILK MIFVHLVIFC

Hedgehog LSRIVIFIEI VGFFIPLILN VTCSSMVLRT LSKPVTLSRS -KINKTKVLR MIFVHLVIFC

Rabbit LSRIVIFIEI VGFFIPLILN VTCSSMVLRT LNKPVTLSRS -KINKTKVLK MIFVHLVIFC

Zebrafish VSKVVVFMET VGFLIPLMIN FICSVKVLQT LRHPETISRG GQLNKAKILR MIVVHLLIFC

Cat wildtype **LSRIVIFIEI VGFFIPLILN VTCSSMVLRT LNKPVTLSRS -KINKTKVLK MIFVHLVIFC**

Cornish Rex ---------- ---------- ---------- ---------- ---------- ----------

....|....| ....|....| ....|....| ....|....| ....|....| ....|....|

245 255 265 275 285 295

Human FCFVPYNINL ILYSLVRTQT FVNCSVVAAV RTMYPITLCI AVSNCCFDPI VYYFTSDTIQ

Alpaca FCFVPYNINL ILYSLMRTQT FVNCSAVTAV RTMYPITLCI AVLNCCFDPI VYYFTSDTIQ

Chimpanzee FCFVPYNINL ILYSLVRTQT FVNCSVVAAV RTMYPITLCI AVSNCCFDPI VYYFTSDTIQ

Elephant FCFVPYNINL ILYSLMRTQT FVNCSVVAAV RTMYPITLCI AVTNCCFDPI IYYFTSDTIQ

Hedgehog FCFVPYNINL ILYSLMRTQT FVNCSAAKAV RTMYPITLCI AVLNCCFDPI VYYFTSDAIQ

Rabbit FCFVPYNINL ILYSLMRTQK FVNCSVVAAV RTMYPITLCI AVSNCCFDPI VYYFTSDTIQ

Zebrafish FCFIPFNVNL VFYTLVRSEV IQNCTVETVV RTIYPIAFCI AVTNCCFDPV IYYFTSETIQ

Cat wildtype **FCFVPYNINL ILYSLMRTQT FVNCSAVTAV RTMYPITLCI AVSNCCFDPI VYYFTSDTIQ**

Cornish Rex ---------- ---------- ---------- ---------- ---------- ----------

....|....| ....|....| ....|....| ....|....| ....|....

305 315 325 335 345

Human NSIKMKNWSV RRSDFRFSEV HGAEN--FIQ HNLQTLKSKI FDNESAA--

Alpaca NSIKMKNWSA RRSDSRFSEV QGTES--FIE HNLQTLKRKI FDNESTIAA

Chimpanzee NSIKMKNWSV RRSDFRFSEV HGAEN--FIQ HNLQTLKSKI FDNESAA--

Elephant NSIKMKNWSV RRSDSRFSEA HGTEN--FIQ HNLQTLKNKI FDNESTI--

Hedgehog NSIKMKSWSS RKSDSRFSEV QGSEN--FIQ HSLQTLKNKI FDNESTIAS

Rabbit NSIKMKNWSV RRNDSRFSEV QGTEN--FIQ HNLQTLKTKI FDNESTI--

Zebrafish NSMKRKSYGV HKNTLNNTID NSDYNGRGTI DKITSLTAKF MIEESTI--

Cat wildtype **NSIKMKNWST RRSDFRFSEV HSTEN--FIQ HNLQTLKSKI FDHES**----

Cornish Rex ---------- ---------- ---------- ---------- ---------

**Figure S3. Protein sequence aligments of *LPAR6* in different species.** *LPAR6* is highly conserved in human, alpaca, chimpanzee, elephant, hedgehog, rabbit, zebrafish, cat wild-type and Cornish Rex. The Cornish Rex mutation causes a frameshift with a premature stop codon occurring at amino acid position number 92. The portion of the Cornish Rex allele that is altered relative to the wildtype is presented in bold.
