# Supplementary material for: To the Root of the Curl: A Signature of a Recent Selective Sweep Identifies a Mutation That Defines the Cornish Rex Cat Breed
Source: PLoS One. 2013 Jun 27;8(6):e67105. doi: 10.1371/journal.pone.0067105 (PMC3694948; doi:10.1371/journal.pone.0067105)
Supplement: File S2 — Supporting tables. Table S1. SNPs with di values above the 99th percentile in the Cornish Rex analysis. Table S2. SNP coordinates in the cat genome with highest di values for Cornish Rex phenotype. Table S3. Consensus details homozygous regions across all Cornish Rex cats. Table S4. Gene symbols and names within the 3 Mb haplotype on chromosome A1. Table S5. SNP identification of LPAR6 in the domestic cats. Table S6. LPAR6 genotyping results in cat breeds with pelage mutations. Table S7. PCR primers for the analysis of LPAR6 in the domestic cat. Table S8. Breeds and populations included in the signatures of selective sweep analyses (di and homozygous regions detection). (DOC) [file pone.0067105.s002.doc]

**Supplementary tables:**

**Table S1**. SNPs with *d_i_* valuesabove the 99^th^ percentile in the Cornish Rex analysis.

| CHR | # of SNPs | Start | end | *d_i_* value |
| --- | --- | --- | --- | --- |
| A1 | 11 | 12525126 | 12981072 | 30.50617645 |
| A1 | 10 | 15558518 | 15967398 | 24.17874457 |
| A1 | 10 | 20087454 | 20493322 | 20.41437252 |
| A1 | 10 | 33544062 | 33947996 | 18.72326042 |
| A1 | 14 | 31513710 | 31934640 | 18.12450972 |
| A1 | 9 | 19557851 | 19953496 | 17.46950542 |
| B4 | 14 | 110006870 | 110487632 | 16.55045967 |
| A1 | 12 | 30522304 | 30974412 | 16.5464056 |
| A1 | 11 | 18551628 | 18983378 | 16.47047911 |
| D1 | 8 | 105095113 | 105464092 | 16.0949603 |
| F2 | 11 | 26526484 | 26971108 | 15.83844715 |
| D1 | 10 | 30521160 | 30978622 | 15.71964975 |
| A1 | 14 | 22024692 | 22475262 | 14.92084685 |
| C2 | 9 | 79574278 | 79959118 | 14.80235972 |
| C2 | 13 | 86546796 | 86976194 | 14.14941384 |
| A1 | 15 | 21544492 | 21992522 | 13.96352495 |
| A1 | 13 | 18015430 | 18452800 | 13.90073812 |
| A1 | 12 | 25043760 | 25417644 | 13.87309463 |
| F1 | 12 | 51519336 | 51988382 | 13.59480347 |
| A1 | 14 | 16000634 | 16489398 | 13.5628446 |
| A1 | 14 | 28527760 | 28961226 | 13.53647965 |
| B4 | 11 | 112506412 | 112980578 | 13.52233492 |
| A1 | 12 | 31019606 | 31459490 | 13.44102618 |
| B2 | 8 | 102210982 | 102476364 | 13.22273957 |
| D1 | 9 | 115028664 | 115452668 | 13.20006623 |
| A1 | 11 | 19048434 | 19499396 | 13.03881878 |
| A1 | 8 | 33082260 | 33493954 | 12.70434562 |
| B4 | 12 | 8047404 | 8448642 | 12.63382866 |
| A3 | 11 | 75548285 | 75997824 | 12.35590724 |
| A1 | 13 | 35563638 | 35993234 | 12.32777602 |
| A1 | 14 | 17523784 | 17986272 | 12.25424841 |
| B2 | 10 | 21041300 | 21403990 | 12.2443723 |
| B3 | 13 | 111016170 | 111498870 | 12.23096968 |
| F2 | 12 | 15547130 | 15968734 | 12.1560509 |
| A1 | 8 | 36020948 | 36449254 | 12.12975819 |
| B4 | 10 | 9513496 | 9996654 | 12.10672105 |
| B4 | 14 | 8502266 | 8980428 | 11.97172733 |
| D1 | 11 | 31014414 | 31495194 | 11.9698938 |
| E1 | 11 | 40509256 | 40980682 | 11.93629278 |
| D2 | 16 | 86531696 | 86993140 | 11.92190305 |
| A1 | 10 | 29539440 | 29912072 | 11.68579092 |
| F1 | 7 | 48016718 | 48475006 | 11.59407038 |
| F1 | 11 | 47513332 | 47990476 | 11.57706762 |
| D2 | 8 | 1518976 | 1980780 | 11.56308061 |

**Table S2**. SNP coordinates in the cat genome with highest *d_i_* values for Cornish Rex phenotype.

| chr | pos | *d_i_* value |
| --- | --- | --- |
| A1 | 12900880 | 60.26919 |
| A1 | 22091468 | 59.63027 |
| A1 | 12679880 | 56.08833 |
| B4 | 1.08E+08 | 55.76666 |
| A1 | 19652010 | 55.53244 |
| A1 | 33635114 | 55.21635 |
| A1 | 30660482 | 52.93686 |
| A1 | 28947524 | 52.814 |
| A1 | 33691387 | 52.30048 |
| B4 | 8746506 | 52.09811 |
| A1 | 15919122 | 51.96823 |
| A1 | 20270322 | 51.95936 |
| A1 | 30624826 | 51.93069 |
| A1 | 18185192 | 51.90192 |
| A1 | 15813236 | 51.4344 |
| B4 | 1.1E+08 | 51.2113 |
| A1 | 31794046 | 50.92541 |
| B4 | 1.1E+08 | 50.37334 |
| A1 | 36928782 | 50.26042 |
| A1 | 12936934 | 50.08801 |

**Table S3**. Consensus details homozygous regions across all Cornish Rex cats.

| SNP1 | SNP2 | bp START | bp END | KB | # SNPs |
| --- | --- | --- | --- | --- | --- |
| chrA1.22661479 | chrA1.23039375 | 18340220 | 18636486 | 296.266 | 8 |
| chrUn22.1093456 | chrA1.25439621 | 20301116 | 23456462 | 3155.35 | 78 |
| chrB4.9049153 | chrB4.10336988 | 7032564 | 8047404 | 1014.84 | 29 |
| chrX.17263927 | chrA1.288521343 | 13681594 | 14743262 | 1061.67 | 27 |
| chrX.29170217 | chrX.30608467 | 23598682 | 24792902 | 1194.22 | 29 |
| chrX.36882317 | chrX.41070417 | 29460574 | 32874238 | 3413.66 | 80 |
| chrX.42347764 | chrX.42846082 | 33879166 | 34269082 | 389.916 | 7 |
| chrX.42902496 | chrX.42961173 | 34325258 | 34351906 | 26.648 | 2 |
| chrX.129205125 | chrX.131284336 | 106241242 | 107777134 | 1535.89 | 40 |

**Table S4**. Gene symbols and names within the 3 Mb haplotype on chromosome A1.

| **GENE SYMBOL** | **GENE NAME** |
| --- | --- |
|  |  |
| GUCY1B2 | guanylate cyclase 1, soluble, beta 2 (pseudogene) |
| RNASEH2B | ribonuclease H2, subunit B |
| DLEU7-AS1 | DLEU7 antisense RNA 1 |
| DLEU7 | deleted in lymphocytic leukemia, 7 |
| DLEU2 | deleted in lymphocytic leukemia, 2 |
| DLEU1 | deleted in lymphocytic leukemia, 1 |
| DLEU2L | deleted in lymphocytic leukemia 2-like |
| MIR15A | microRNA 15a |
| MIR16-1 | microRNA 16-1 |
| KCNRG | potassium channel regulator |
| TRIM13 | tripartite motif containing 13 |
| SPRYD7 | SPRY domain containing 7 |
| KPNA3 | karyopherin alpha 3 |
| KPNA4 | karyopherin alpha 4 |
| EBPL | emopamil binding protein-like |
| ARL11 | ADP-ribosylation factor-like 11 |
| RCBTB1 | regulator of chromosome condensation (RCC1) and BTB (POZ) domain containing protein 1 |
| PHF11 | PHD finger protein 11 |
| SETDB2 | SET domain, bifurcated 2 |
| CAB39L | calcium binding protein 39-like |
| CDADC1 | cytidine and dCMP deaminase domain containing 1 |
| MLNR | motilin receptor |
| FNDC3A | fibronectin type III domain containing 3A |
| CYSLTR2 | cysteinyl leukotriene receptor 2 |
| RCBTB2 | regulator of chromosome condensation (RCC1) and BTB (POZ) domain containing protein 2 |
| RB1 | retinoblastoma 1 |
| **P2RY5*** | lysophosphatidic acid receptor 6 |
| ITM2B | integral membrane protein 2B |
| MED4 | mediator complex subunit 4 |
| NUDT15 | nudix (nucleoside diphosphate linked moiety X)-type motif 15 |
| SUCLA2 | succinate-CoA ligase, ADP-forming, beta subunit |

* strong candidate gene

**Table S5. SNP identification of *LPAR6* in the domestic cats.**

|  |  |  | **Nucleotide Sequence** | | | |
| --- | --- | --- | --- | --- | --- | --- |
|  |  |  | **5’UTR -194** | **5’UTR -72** | **E5 63** | **E5 250-253** |
| **Breed** | **Hair Phenotype** | **No.** | **G/A** | **C/T** | **C/T** | **TTTG/del** |
| Cornish Rex | Curly | 9 | G | C | C | Del |
| American Wirehair | Wire | 1 | G | C/T | C | TTTG |
| Burmese | Straight | 1 | G | T | C | TTTG |
| Devon Rex | Curly | 1 | G | C | C | TTTG |
| German Rex | Curly | 1 | G | C | C | Del |
| LaPerm* | Curly | 1 | A/G | T | T | TTTG |
| Peterbald | Hairless | 1 | G | C | C | TTTG |
| Random Bred | Straight | 1 | G | C/T | C/T | TTTG |
| Selkirk Rex | Curly | 1 | G | C/T | C | TTTG |
| Siamese | Straight | 1 | G | C | C | TTTG |
| Sphynx | Hairless | 1 | G | C | C | TTTG |
| Tennessee Rex | Curly | 1 | G | C/T | C | TTTG |
| Ural Rex* | Curly | 1 | A/G | C | C | TTTG |
| **Total** |  | **21** |  |  |  |  |

*Further investigation is required for this polymorphism in the 5’ UTR of *P2RY5*, as it cannot be exclude as causative for the two dominant curly hair conditions within the two breeds.

**Table S6.** *LPAR6* genotyping results in cat breeds with pelage mutations.

| Breed | Hair Phenotype | No.* | *LPAR6^wt/wt^* | *LPAR6^wt/del^* | *LPAR6^del/del^* |
| --- | --- | --- | --- | --- | --- |
| Cornish Rex | Curly | 39 | 0 | 0 | 39 |
| American Wirehair | Wired | 3 | 3 | 0 | 0 |
| Devon Rex | Curly | 7 | 7 | 0 | 0 |
| German Rex | Curly - straight | 4 | 0 | 2 | 2 |
| La Perm | Curly | 6 | 6 | 0 | 0 |
| Peterbald | Hairless | 4 | 4 | 0 | 0 |
| Random Bred | Straight | 8 | 8 | 0 | 0 |
| Selkirk | Curly | 6 | 6 | 0 | 0 |
| Sphynx | Hairless | 7 | 7 | 0 | 0 |
| Tennessee Rex | Curly | 4 | 4 | 0 | 0 |
| Ural Rex | Curly | 2 | 2 | 0 | 0 |
| Total |  | 90 | 47 | 2 | 41 |

^*^ Includes the 21 samples sequenced and presented in supplementary Table 1.

**Table S7.** PCR primers for the analysis of *LPAR6* in the domestic cat.

|  | Primer Sequence | | Product |
| --- | --- | --- | --- |
| Region | Forward Primer 5’-3’ | Reverse Primer 5’-3’ | Size (bp) |
| Exon 5 | TAATCTAACAGGCTGGCTTCA | GCAGGCTTCTGAGGCATTGTT | 825 |
|  | CCCATTTAAGTCAAAGACTCT | TAAGGTCTGTAGGTTATGTTG | 640 |
| cDNA* | TCCTGTTGAAGAACCAAGTGG | GCAGGCTTCTGAGGCATTGTT* |  |
|  | CCCATTTAAGTCAAAGACTCT* | TAAGGTCTGTAGGTTATGTTG* |  |
|  | AACTGGTCTACTAGGAGAAGT | poly T |  |
| Genotype | F- GCGGTAACAATTCACAGGACCCTCAAAGTGCGAAATGAAACT | | |
|  | R - GCAGGCTTCTGAGGCATTGTT | | |

*Same primer used for genomic and cDNA

**Table S8**. Breeds and populations included in the signatures of selective sweep analyses (d_i_ and homozygous regions detection).

| Breed | N |
| --- | --- |
| Abyssinian | 18 |
| Birman | 20 |
| Burmese | 20 |
| Cornish | 12 |
| Eastern random bred | 19 |
| Egyptian Mau | 15 |
| Japanese bobtail | 13 |
| Maine coon | 20 |
| Norwegian forest cat | 18 |
| Persian | 21 |
| Ragdoll | 12 |
| Siamese | 19 |
| Turkish van | 19 |
| Western random bred | 21 |
| Total | 247 |
